# CpG promoter methylation of the ALKBH3 alkylation repair gene in breast cancer

**DOI:** 10.1186/s12885-017-3453-8

**Published:** 2017-07-05

**Authors:** Olafur Andri Stefansson, Stefan Hermanowicz, Jasper van der Horst, Holmfridur Hilmarsdottir, Zuzanna Staszczak, Jon Gunnlaugur Jonasson, Laufey Tryggvadottir, Thorkell Gudjonsson, Stefan Sigurdsson

**Affiliations:** 1 Cancer Research Laboratory, Biomedical Center, Vatnsmyrarvegur 16 (4th floor), 101 Reykjavik, Iceland; 2 Faculty of Medicine, University of Iceland, Vatnsmyrarvegur 16 (4th floor), 101 Reykjavik, Iceland; 3 Department of Biochemistry and Molecular Biology, Biomedical Center, Vatnsmyrarvegur 16 (5th floor), 101 Reykjavik, Iceland; 4 Icelandic Cancer Registry, Skogarhlid 8, Reykjavik, Iceland; 5 Department of Pathology, Landspitali University Hospital, Reykjavik, Iceland

**Keywords:** Breast cancer, ALKBH3, Epigenetics, DNA methylation, DNA repair, Alkylation, Prognosis

## Abstract

**Background:**

DNA repair of alkylation damage is defective in various cancers. This occurs through somatically acquired inactivation of the MGMT gene in various cancer types, including breast cancers. In addition to MGMT, the two *E. coli* AlkB homologs ALKBH2 and ALKBH3 have also been linked to direct reversal of alkylation damage. However, it is currently unknown whether ALKBH2 or ALKBH3 are found inactivated in cancer.

**Methods:**

Methylome datasets (GSE52865, GSE20713, GSE69914), available through Omnibus, were used to determine whether ALKBH2 or ALKBH3 are found inactivated by CpG promoter methylation. TCGA dataset enabled us to then assess the impact of CpG promoter methylation on mRNA expression for both ALKBH2 and ALKBH3. DNA methylation analysis for the ALKBH3 promoter region was carried out by pyrosequencing (PyroMark Q24) in 265 primary breast tumours and 30 proximal normal breast tissue samples along with 8 breast-derived cell lines. ALKBH3 mRNA and protein expression were analysed in cell lines using RT-PCR and Western blotting, respectively. DNA alkylation damage assay was carried out in cell lines based on immunofluorescence and confocal imaging. Data on clinical parameters and survival outcomes in patients were obtained and assessed in relation to ALKBH3 promoter methylation.

**Results:**

The ALKBH3 gene, but not ALKBH2, undergoes CpG promoter methylation and transcriptional silencing in breast cancer. We developed a quantitative alkylation DNA damage assay based on immunofluorescence and confocal imaging revealing higher levels of alkylation damage in association with epigenetic inactivation of the ALKBH3 gene (*P* = 0.029). In our cohort of 265 primary breast cancer, we found 72 cases showing aberrantly high CpG promoter methylation over the ALKBH3 promoter (27%; 72 out of 265). We further show that increasingly higher degree of ALKBH3 promoter methylation is associated with reduced breast-cancer specific survival times in patients. In this analysis, ALKBH3 promoter methylation at >20% CpG methylation was found to be statistically significantly associated with reduced survival (HR = 2.3; *P* = 0.012). By thresholding at the clinically relevant CpG methylation level (>20%), we find the incidence of ALKBH3 promoter methylation to be 5% (13 out of 265).

**Conclusions:**

ALKBH3 is a novel addition to the catalogue of DNA repair genes found inactivated in breast cancer. Our results underscore a link between defective alkylation repair and breast cancer which, additionally, is found in association with poor disease outcome.

**Electronic supplementary material:**

The online version of this article (doi:10.1186/s12885-017-3453-8) contains supplementary material, which is available to authorized users.

## Background

Epigenetics can be described as the study on mechanisms by which changes in phenotype are established and stably maintained following cellular divisions without involving any changes in genotype. The fundamental unit of chromatin is the nucleosome representing a short stretch of DNA wrapped around a protein complex consisting of histone variants arranged as octamers [1]. Nucleosome occupancy over regulatory regions in DNA associates with transcriptional activity as densely occupied regions are poorly accessible to transcription factors [2]. The degree of nucleosome occupancy, or chromatin packaging, is regulated by the use of so-called epigenetic marks of which the best studied is undoubtedly DNA methylation involving the addition of methyl groups to the 5-position on cytosine bases (5-meC) where cytosine is followed by guanine, i.e. so-called CpG sites [3]. Methyl-Binding Domain-containing proteins recognize and bind to methylated CpGs and recruit histone modifiers to mediate or maintain repressed chromatin structure [3].

In cancer cells, the epigenome is frequently disrupted - characterized by global loss in repressive marks and localized modifications over regulatory elements [4]. It is now known that genes functionally involved in shaping the epigenome of human cells are recurrently mutated in breast cancer and various other cancers, e.g. MLL3, MLL2, ARID1A and SETD2 [5]. The discoveries of recurrent mutations in epigenetic genes provided a convincing link between disruptions in the epigenome and the development of cancer. In addition to this, earlier observations had already established repressive epigenetic marks over regulatory regions of known tumor suppressor genes in cancer cells. In breast cancer, this catalogue includes CpG promoter methylation of BRCA1, RAD51C, FOXC1, RUNX3 and L3MBTL4 [6–10]. Of these, the BRCA1 gene is well established as a cancer predisposition gene where germline mutations are found in association with greatly increased risk for breast and ovarian cancer [11]. Other high-risk breast cancer susceptibility genes such as BRCA2, PALB2, BARD1, FANCM, ATM, CHEK2 and TP53, however, are not found epigenetically silenced [12, 13].

The onset of a subset of breast cancers is strongly tied to defective repair of DNA double-stranded breaks by homologous recombination [14, 15]. In recent years, several researchers have reported loss of MGMT in breast cancer thereby implicating defective alkylation repair in breast cancer development [16–18]. The MGMT gene has an important role in removing cytotoxic adducts from O(6)-guanine in DNA [19]. Proficiency for alkylation repair is critical to protect the cell against accumulation of genetic mutations. Indeed, recent cancer genome sequencing studies have revealed a profound impact from treatment with alkylating agent temozolomide leading to a specific mutational pattern [20]. This observation provides a solid link between alkylation damage and the formation of genetic mutations.

Alkylating agents are by-products of normal cellular metabolism as well as being ubiquitous in the environment [21]. In addition to MGMT, at least two other genes are known to be involved in direct reversal of alkylation damage, i.e. the *E. coli* AlkB homologs ALKBH2 and ALKBH3 both oxidative demethylases involved in the repair of 1-methyladenine and 3-methylcytosine [21]. It is currently unknown whether ALKBH2 or ALKBH3 undergo epigenetic silencing in cancer. In this study, we demonstrate that the ALKBH3 gene, not ALKBH2, is found recurrently silenced in breast cancer by epigenetic events which, furthermore, defines a group of patients with dramatically reduced survival.

## Methods

### Study cohort

DNA samples were derived from primary breast tumors (*n* = 265) and adjacent normal breast tissue (*n* = 30). The normal breast tissue was obtained from non-tumorous regions of the breast. The DNA was previously isolated from freshly frozen tissue following a standard protocol based on phenol-chloroform (+proteinase K) extraction. RNA samples were available for a subset of the tumor (*n* = 36) and normal breast (*n* = 10) tissue samples isolated using Tri-Reagent (Thermo Fisher Scientific). Clinical parameters, including tumor size, nodal status, histological grade along with disease-specific follow-up times were obtained from the nationwide Icelandic Cancer Registry [22]. This work was carried out according to permits from the Icelandic Data Protection Commission (2006050307) and Bioethics Committee (VSNb2006050001/03–16). Informed consent (written) was obtained from all patients.

The cell lines used in this study were obtained from the American Type Culture Collection. The cells were cultured in DMEM (CAMA-1, MDAMB-468, MCF-7, MCF-10A, MDAMB-231 and SKBr-3) or RPMI (HCC-38, Bt-474) with added 10% serum (+penicillin/streptomycin). DNA and RNA was extracted in parallel from the cell lines using Qiagen’s AllPrep DNA/RNA/miRNA Universal kit (80224; Qiagen).

### DNA methylation analyses

Bisulfite conversion was carried out using the EZ-96 DNA Methylation-Gold kit from Zymo Research (D5008). We carried out the bisulfite conversion for 16 cycles of {95 °C for 30 s and 50 °C for 1 h} to then hold at 4 °C until samples were added to the DNA columns for completing the conversion following the manufacturer’s guidelines (Zymo Research).

The PyroMark Assay Design 2.0 software was used to design primers for the analysis of ALKBH3 promoter methylation. The following primer sequences were used: 5′-(Btn)-GTGGGATTATTAGGATTGAGGATT-3′ (5-biotin labelled) and 5′-CTCCAACAACTCCCAATCAC-3′. The pre-amplification PCR reaction was carried out using a hot-start PCR polymerase (Immolase DNA polymerase from Bioline; Bio-21,047). The PCR conditions were as follows: 96 °C for 10 min, 45 cycles of (96 °C for 30s, 60 °C for 30s and 72 °C for 30s) followed by 15 min hold at 72 °C and then 4 °C. The PCR products were then captured using streptavidin coated agarose beads (Streptavidin Sepharose High Performance 34 μm beads, GE Healthcare) under denaturing conditions to obtain single-stranded DNA. The pyrosequencing reaction was then carried out using the PyroMark Q24 machine (Qiagen) and PyroMark Gold-Q24 Reagents kit (Qiagen) using the following sequencing-primer: 5′-ACATCAAACACTTCCT-3′.

CpG methylation for three CpG’s were assessed (−58, −53 and −50 bp upstream of the TSS (p1) given the FANTOM5 promoterome database) [23]. The output data (obtained from PyroMark Q24 sequencing reactions), representing percent methylated cytosines over each of these three CpGs, was averaged for each sample analysed. This yielded a single measure representing a proxy for CpG methylation levels over the ALKBH3 promoter region. The statistical analysis of paired tumor and normal breast tissue samples made use of a paired Wilcoxon’s test using the wilcox.test function in R.

### Expression analyses in normal breast tissue, tumors and cancer cell lines

RNA was extracted from tumors and normal breast tissues using Tri-Reagent (Thermo Fisher Scientific). The RNA derived from cell lines was isolated in a simultaneous DNA/RNA isolation procedure using the Qiagen’s Allprep kit (Qiagen). Reverse transcription was carried out using High-Capacity cDNA Reverse-Transcription Kit (Thermo Fisher Scientific). ALKBH3 expression was quantitatively analysed by the SYBR green method using a real-time PCR (RT-PCR) machine (Applied Biosystems 7500). HPRT1 expression was used to normalize the expression data by computing the difference in Ct as follows: 2^-(ALKBH3 Ct – HPRT1 Ct)^. The primers used for ALKBH3 were: 5′-AGCCACGAGTGATTGACAGAG-3′ and 5′- ACAAACAGACCCTAGATACACCT-3′, and for HPRT1: 5′- CCTGGCGTCGTGATTAGTGAT-3′ and 5′-AGACGTTCAGTCCTGTCCATAA-3′. The Spearman’s rank test for correlation was carried out using the cor.test function in R to assess the association between CpG methylation and expression.

Proteins were extracted from cell lines at 80% confluency using the EBC lysis buffer and measured at 490 nm using a spectrophotometer. The samples were denatured and electropherized using a 10% gel followed by transfer to PVDF membrane. The primary antibody (Millipore anti-ALKBH3 rabbit polyclonal 09–882) was used at 1:500 dilution overnight at 4 °C followed by washing with PBS-Tween. The secondary antibody (Santa Cruz donkey anti-rabbit IgG-HRP, sc-2313) was used at 1:10,000 dilution. The membrane was developed with ECL (Pierce ECL Plus Western Blotting Substrate, Thermo Scientific 32,132) and detected in a ImageQuant LAS4000. The β-actin primary antibody (MAB1501R; Millipore) was used at a dilution of 1:20,000 with secondary HRP-rabbit antibody anti-mouse IgG used at 1: 10,000 dilution (61–6020; Invitrogen).

### DNA alkylation damage detection

CAMA1 and MDA-MB-468 were grown on coverslips and fixed with freshly prepared 4% para-formaldehyde solution for 15 min. After fixation, cells were treated with 1.5 M HCL for 20 min, to gain access to single stranded DNA, followed by a 2-min treatment with Sodium Borate (pH 8.5) to neutralize the acid. After permabilization (5 min, 0.2% TritonX) and 1 h of blocking (DMEM (Gibco) with 10% FBS (Gibco)) cells were stained with antibodies against 3-methylcytosine (3meC) (rabbit, Active Motif, 61111) and 5-methylcytosine (5meC) (mouse, abcam, ab10805) for 1 h at room temperature. Both antibodies were diluted 1:250 in blocking buffer. Next, samples were incubated with secondary antibodies, Alexa-Fluor 488 goat anti rabbit (A11008, Thermo Fisher Scientific) and Alexa-Fluor 555 goat anti-mouse (A21422, Thermo Fisher Scientific), diluted in blocking buffer (1:1000) for 1 h. Nuclear DNA was stained by DAPI (SIGMA, D9542). The DAPI stain was added directly to the secondary antibody solution (diluted 1:5000). After drying, the coverslips were mounted on glass slides using Fluoroshield (SIGMA, F6182) mounting medium.

Images were acquired using the FV1200 Olympus inverted confocal microscope. Dual colour confocal images were acquired with standard settings using laser lines 488 nm and 543 nm for excitation of Alexa Fluor 488 and Alexa Fluor 568 dyes, respectively. Nuclear DAPI staining was imaged using excitation by the 405 nm laser. For each condition 10 images were randomly acquired with the 20X/0.75 objective and imported into CellProfiler for downstream image analysis. For each data point, 400–600 nuclei (identified by DAPI staining) were analysed for 3meC and 5meC nuclear intensity (mean integrated intensity).

The 3meC and 5meC values presented in Fig. 2d are based on four independent staining experiments. The Wilcoxon’s rank sum hypothesis test was used to assess differences in 3meC and 5meC values (in R 3.1.0).

### Tissue microarrays (TMAs)

Estrogen receptor (ER), progesterone receptor (PR) and HER-2 expression were previously analysed on tumors by immunohistochemistry (IHC) on tissue microarrays (TMAs) [24]. The TMAs were constructed as previously described (Stefansson et al. 2009). Immunohistochemistry (IHC) was then applied using 4 μm thick TMA sections using the following anti-bodies: anti-ER (1D5; DAKO), anti-PR (PgR 636; DAKO) and anti-HER2 (HercepTest Kit; DAKO). ER and PR were scored positive given any visible nuclear staining in more than 1% of tumor cell nuclei. HER-2 positivity was defined as score of 3+ according to criteria provided by the anti-body manufacturer.

### Informatics and statistical analyses

Information on CpG methylation over the promoter region of ALKBH2 and ALKBH3 was obtained from pre-existing methylome analyses published by Stefansson et al. (GSE52865), Dedeurwaerder et al. (GSE20713) and Teschendorff et al. (GSE69914) available through the Omnibus repository at NCBI’s website (www.ncbi.nlm.nih.gov/gds/) [7, 25, 26]. The normalized data were extracted from the SOFT formatted files using the GEOquery package in R and analysed by comparing normal breast tissue samples and breast cancers. This was carried out using the Student’s t-test on M–values computed using M_i_ = log_2_(B_i_ / (1-B_i_)) where B represents the β-value coupled with the Benjamini-Hochberg adjustment procedure to account for multiple hypothesis testing making use of the p.adjust function in R. The multiple hypothesis adjustment accounted for the total number of CpGs represented on the array platform, i.e. adjusting for the entire >450 thousand CpGs in GSE52865 and GSE69914 and >27 thousand CpGs in GSE20713.

DNA methylation (450 K Infinium) and RNAseq (V2) level 3 data were downloaded from the Cancer Genome Atlas data repository (http://cancergenome.nih.gov/) [27]. Firstly, the analysis of differential ALKBH3 mRNA expression levels between normal breast tissue and breast cancers was carried out using the Wilcoxon’s rank sum hypothesis test taking into account adjustment for multiple hypothesis testing including the entire set of >20 thousand genes included in the RNAseqV2 dataset. This was carried out using the Benjamini-Hochberg (BH) procedure through the p.adjust function in R. Secondly, the relation between CpG methylation for each site represented over either ALKBH2 and ALKBH3 were studied with respect to ALKBH3 mRNA expression using Spearman’s correlation analysis and, as before, with genome-wide adjustment of the *P*-values using the BH procedure to account for multiple hypothesis testing.

Information on epigenetic marks for the ALKBH3 promoter region in variant human mammary epithelial cells (vHMEC) was obtained from the Roadmap Epigenomics browser (egg2.wustl.edu/roadmap/web_portal/) [28]. This includes information on ALKBH3 expression based on RNA sequencing and chromatin marks based on ChIP-seq along with data on chromatin accessibility based on DNA sequencing. Data on CpG methylation for the vHMEC cells was derived from methylCRF computational analysis using MeDIP-seq and MRE-seq data to infer whole-genome 5-methylcytosine states as carried out and provided by the Roadmap Epigenomics project [28].

Information on nucleotide positions for ALKBH3 gene structure (introns and exons) was downloaded from Ensembl (GRCh37 browser; HG19). Data on transcriptional start site (TSS) and CpG islands were obtained from the FANTOM5 promoterome [23]. Using the UCSC genome browser, the chromStart/chromEnd fields in the hg19.cpgIslandExt table provided the CpG island positional information. The R statistical software (R 3.1.0) was then used to graphically represent the ALKBH3 promoter with respect to the TSS, 1st Exon and CpG island.

The association between ALKBH3 promoter methylation and subtype-specific markers was assessed using wilcoxon’s rank sum hypothesis testing (wilcox.test in R). The chi-squared test was used to assess the association between tumor subtype classification, histological grade, tumor size and nodal status (chisq.test in R). Differences in breast cancer-specific patient survival with respect to ALKBH3 methylation in tumor tissue was assessed using the log-rank hypothesis test (survdiff function in R). Cox’s proportional hazards regression model was use for multivariate analysis of survival (coxph function in R). The cox.zph function in R was applied to assess the assumptions of the regression model with respect to proportionality over time.

## Results

### Methylome analyses identify ALKBH3 as a target of CpG promoter methylation in breast cancer

**Fig. 1:**
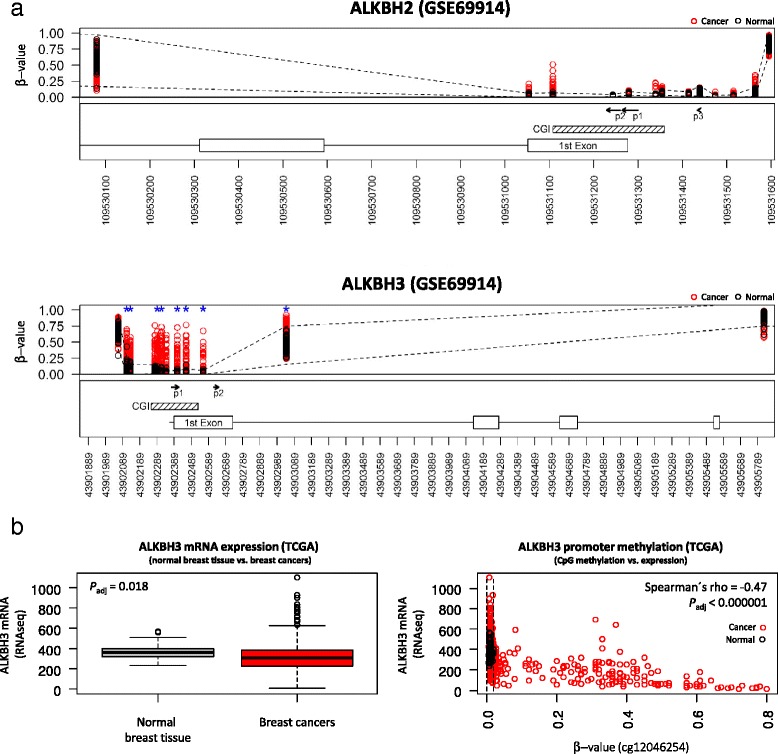
CpG methylation over the promoter region ALKBH2 and ALKBH3 in breast cancers and normal breast tissue samples. **a** CpG methylation states obtained from GSE69914 over ALKBH2 (upper panel) and ALKBH3 (lower panel) in normal breast tissue samples and breast cancers in *black* and *red*, respectively. The y-axis represents β (beta)-values (reflecting the degree of 5-cytosine methylation) for CpG’s included on the Infinium arrays located within or proximal to the promoter region for ALKBH2 and ALKBH3 arranged on the x-axis according to nucleotide position. The dashed lines (in *black*) represent the upper and lower 99% confidence intervals for the distribution of β-values in normal breast tissue samples – thus displaying the “normal range” for each of the CpGs analysed. Differentially methylated CpGs between breast cancers and normal breast tissues are indicated by *blue* asterisk marks taking into account correction for multiple hypothesis testing including all CpGs represented on the 450 K array (P_adj_ < 0.001). FANTOM5 regions for ALKBH2 and ALKBH3 are shown as arrows indicating transcription start sites (TSS) where p1 represents the major TSS (while p2 and p3 are less prominently used as TSS). Additionally, the location of UCSC defined CpG islands (CGI; strikethrough patterned boxes) and the 1st Exon for each of the two genes are labelled. UCSC defined CpG islands from the UCSC genome table browser. **b** Left panel; ALKBH3 mRNA expression levels by RNA sequencing (RNAseq) obtained from the TCGA dataset analysed with respect to normal breast tissue samples compared with breast cancers. These differences reflect generally lowered expression levels in breast cancers compared with normal breast tissue samples. The *P*-value indicated in the upper-left corner was derived from Wilcoxon’s hypothesis testing after adjusting for multiple hypotheses accounting for >20 thousand protein-coding genes represented in the RNAseq dataset (P_adj_ = 0.018). The right panel displays the topmost significant CpG (ranked according to the adjusted *P*-value), i.e. cg12046254, illustrating the relation between ALKBH3 mRNA expression (y-axis) and CpG promoter methylation (x-axis). Again, the dashed lines (in *black*) represent the lower and upper 99% confidence limits for the normal breast tissue samples – reflecting the “normal range” of 5-methylcytosine levels for this particular CpG (cg12046254). The *P*-value indicated in the top-right corner, based on Spearman’s rho correlation analysis, was highly significant even after adjustment for multiple hypothesis testing

By making use of methylome data for breast cancers and normal breast tissues, we specifically asked whether aberrant CpG methylation events are found over the promoter region of either ALKBH2 or ALKBH3. To achieve this, we used datasets available through Omnibus including those published by Stefansson et al. (GSE52865), Dedeurwaerder et al. (GSE20713) and Teschendorff et al. (GSE69914) [7, 25, 26]. The analysis of these datasets consistently identify aberrant CpG methylation over the ALKBH3 gene promoter in breast cancers. Figure 1a illustrates this finding where statistically significant CpG methylation events are seen over the ALKBH3 promoter region (P_adj_ < 0.001). In contrast, differential methylation between breast cancer and normal breast tissue was not identified over the promoter region of ALKBH2 (Fig. 1a).

We used the Cancer Genome Atlas dataset to assess the impact of ALKBH3 promoter methylation on mRNA expression levels. Firstly, these data provide additional support for differential methylation over the ALKBH3 promoter region between normal breast tissue and tumours (data not shown). Secondly, significantly lower expression levels of ALKBH3 mRNA were seen in breast cancers compared with normal breast tissue samples. This finding holds statistically significant after adjusting for multiple hypothesis testing taking into account the entire >20 thousand protein-coding genes in the RNAseqV2 TCGA dataset (Wilcoxon’s rank sum test; *P*_adjusted_ = 0.018) as shown in Fig. 1b (left panel). Thirdly, the majority of the CpGs identified as differentially methylated are also found significantly associated with down-regulation of ALKBH3 mRNA levels in breast cancers (Fig. 1b, right panel; Additional file 1). The topmost significant CpG derived from this analysis (Spearman’s rho = −0.47; P_adj_ < 0.00001) is represented by cg12046254 shown in Fig. 1b (right panel).

### Epigenetics and expression of ALKBH3 in normal breast epithelial cells

**Fig. 2:**
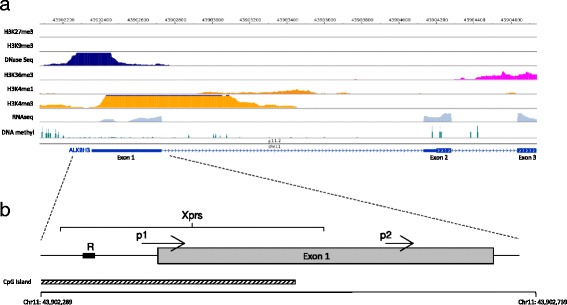
The ALKBH3 gene promoter region. **a** The ALKBH3 gene promoter is transcriptionally active in variant human mammary epithelial cells (vHMEC) based on available data from the Roadmap Epigenetic Consortium (Roadmap Epigenomics Consortium, Nature 2015). Data for vHMEC are shown here for markers associated with active transcription (H3K4me3 and H3K36me3) together with repressive markers (H3K27me3, H3K9me3, H3K4me1 and 5-methylcytosine (DNA methyl)). Additionally, DNAse and RNA sequencing results from vHMEC cells are shown – reflecting chromatin accessibility and mRNA expression, respectively. **b** The ALKBH3 gene promoter region is shown with respect to the FANTOM5 transcription start site (TSS) as arrows p1 and p2 along with the 1st exon and the promoter-associated CpG island (UCSC defined). The CpG methylation assay for ALKBH3 was designed to include CpG sites proximal to the TSS and the regions selected is indicated by a *black* box (labelled R) covering three closely spaced CpG dinucleotides found -50, −53 and -58 bp upstream of the major TSS (p1 region in FANTOM5). Additionally, the region where statistically significant associations were revealed between CpG methylation and loss of expression for the ALKBH3 gene in tumors is marked out and labelled for expression as “Xprs” (see further information in Additional file 1)

Information on epigenetic regulation and expression of the ALKBH3 gene in human mammary epithelial cells (vHMEC) is displayed in Fig. 2a demonstrating transcriptionally active chromatin configuration over the promoter region. This can be seen in DNase-seq signal peaks found upstream of the promoter and extending into the first exon together with active histone markings, i.e. H3 lysine 4 tri-methylation (H3K4Me3) localized over the first exon and H3 lysine 36 tri-methylation (H3K36Me3) over the gene body region collectively indicating open chromatin and active transcription (Fig. 2a). Notably, the H3K4Me3 activation marks are found in the absence of repressive H3 lysine 4 mono-methylation (H3K4me1), H3 lysine 27 tri-methylation (H3K27Me3) and H3 lysine 9 tri-methylation (H3K9Me3) as shown in Fig. 2a. Indeed, the expression data (RNAseq track) shows clear signals from all ten exons of the ALKBH3 gene – the first three exons are shown in Fig. 2a.

In agreement with transcriptionally active chromatin configuration, the ALKBH3 gene promoter region and first exon are lacking of repressive CpG methylation marks (DNA methyl track in Fig. 2a). The ALKBH3 promoter is associated with a CpG island (spanning a region from 43,902,254 bp to 43,902,528 bp) extending into the first exon (Fig. 2b) and, indeed, the entire CpG island (CGI) is found unmethylated in the vHMEC breast epithelial cells. The CGI was identified from the UCSC genome table browser defined as regions of at least 200 bp where the GC content is at least 50%. The ALKBH3 promoter associated CGI was found to be 274 base pairs in length.

We designed a DNA methylation pyrosequencing assay to carry out CpG methylation analysis for the ALKBH3 gene promoter region (Fig. 2b). The DNA region assayed, labelled R in Fig. 2b, includes three CpG sites found −50, −53 and -58 bp upstream of the major TSS (FANTOM5 element p1). In this way, the assay was designed to reflect ALKBH3 promoter methylation status which we then applied across 303 DNA samples, i.e. 8 breast-derived cell lines, 30 normal breast tissue samples and 265 primary breast tumors.

### ALKBH3 epigenetic repression found in two breast cancer cell lines

**Fig. 3:**
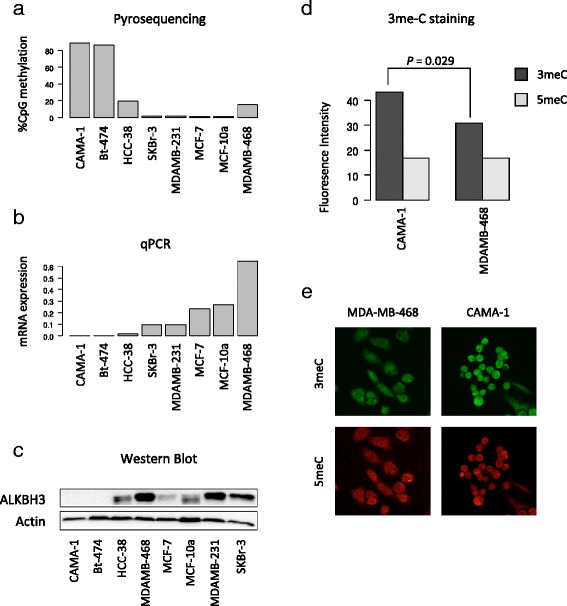
Epigenetic silencing of ALKBH3 and accumulation of 3-methylcytosine damage. **a** CpG methylation analysis for the ALKBH3 gene promoter by pyrosequencing and (**b**) ALKBH3 mRNA expression analysis by qPCR in breast cancer cell lines (CAMA1, Bt-474, HCC-38, SKBr-3, MDA-MB-231, MCF-7 and MDA-MB-468) and a mammary epithelial cell line derived from a fibrocystic lesion of the breast (MCF10A). The association between ALKBH3 promoter methylation and mRNA expression was found to be statistically significant (Spearman’s rho = −0.73; *P* = 0.039). **c** ALKBH3 protein expression analysed by western blotting using the same panel of breast-derived cell lines revealing lack of expression in two cell lines (CAMA-1 and Bt-474). Actin protein expression is shown for comparison. **d** Immunofluorescent staining for 3-methylcytosine and 5-methylcytosine in CAMA-1 (ALKBH3 methylated) and MDA-MB-468 (ALKBH3 unmethylated). The *P*-value shown was derived from Wilcoxon’s rank sum testing for differences between CAMA-1 and MDA-MB-468 with respect to intensity for 3-methylcytosine. As expected, no statistically significant differences were found with respect to overall 5-methylcytosine intensity levels between CAMA-1 and MDA-MB-468. **e** Representative images showing 3-methylcytosine (*green*) and 5-methylcytosine (*red*) fluorescence staining in CAMA-1 and MDA-MB-468

Out of the eight breast-derived cell lines analysed with respect to ALKBH3 promoter methylation, seven were derived from breast tumors (CAMA-1, Bt-474, HCC-38, SKBr-3, MDA-MB-231, MCF-7, MDA-MB-468) and one was derived from a fibrocystic breast lesion (MCF-10A). We identified ALKBH3 promoter methylation in two of the seven breast cancer cell lines, i.e. in CAMA-1 and Bt-474 (Fig. 3a). The MCF10A cell line, often used to reflect normal breast epithelial cells, was clearly unmethylated over the ALKBH3 promoter.

ALKBH3 mRNA expression was not detected in either CAMA-1 or Bt-474 whereas all other cell lines showed ALKBH3 mRNA expression (Fig. 3b). The association between ALKBH3 promoter methylation and mRNA expression was found to be statistically significant (Spearman’s rho = −0.73; *P* = 0.039). Further, complete loss of ALKBH3 protein expression was seen only in the two cell lines showing ALKBH3 promoter methylation, i.e. CAMA-1 and Bt-474 (Fig. 3c).

ALKBH3 has been reported to catalyse the removal of 3-methylcytosine (3meC) on single stranded DNA [29]. In line with that, RNAi mediated knockdown of ALKBH3 has previously been shown to cause an increase in 3meC levels on single-stranded DNA [30]. To determine the functional impact of ALKHB3 promoter methylation, we developed a novel imaging-based assay for the quantification of 3meC on single stranded DNA. Using this method we confirmed previous findings [30] demonstrating increased formation of 3meC damage following siRNA knock-down of ALKBH3 (Additional file 2). Further, we show statistically significant differences in 3meC damages between ALKBH3 deficient cell line CAMA-1 compared with ALKBH3 expressing cell line MDA-MB-468 (*P* = 0.029), see Fig. 3d and e. For reference, we show that 5meC intensities are not significantly different between CAMA-1 and MDA-MB-468 (*P* = 0.69). This indicates that, comparable to knockdown of ALKBH3 [30], epigenetic inactivation of ALKBH3 results in a higher burden of 3meC, most likely because of less efficient repair of alkylation damage.

### CpG promoter methylation and expression of the ALKBH3 gene in normal and primary breast tumor samples

**Fig. 4:**
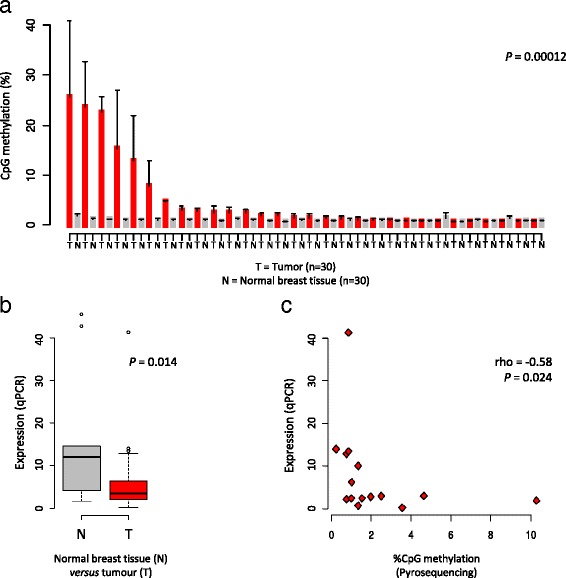
ALKBH3 promoter methylation in primary breast tumors and adjacent normal breast tissue. **a** Differential CpG methylation over the ALKBH3 promoter is observed in a subset of primary breast tumors. The tumor (T; red coloured bars) and normal breast tissue (N; grey coloured bars) samples are matched, i.e. derived from the same individual and arranged side-by-side on the x-axis with ALKBH3 promoter methylation for each sample represented as a bar. The standard deviation is shown (line extensions from the bars). The *P*-value shown was derived from a paired Wilcoxon’s hypothesis test (*P* = 0.00012). **b** Box and whisker plot of ALKBH3 mRNA expression in normal breast tissue samples and primary breast tumors. The *P*-value shown was derived from a Wilcoxon’s hypothesis test (*P* = 0.014). **c** ALKBH3 promoter methylation data plotted on x-axis and mRNA expression data on y-axis for primary breast tumors. The *P*-value shown was derived from Spearman’s correlation testing (Spearman’s rho = −0.577; *P* = 0.024)

Out of the 265 primary tumors analysed with respect to ALKBH3 promoter methylation, a subset of 30 tumor samples were matched with adjacent normal breast tissue samples from the same patients – thereby enabling assessment of differential methylation in paired samples. This analysis reveals clear differences in CpG methylation over the ALKBH3 promoter region between primary tumors and normal breast tissue from the same 30 individuals (Fig. 4a).

This analysis further shows that, in normal breast tissue, the mean methylation levels over the ALKBH3 promoter region is approximately 1.0% (99%CI: 0.12–1.9%). Deviations from the 99% confidence interval for ALKBH3 promoter methylation levels in normal breast tissue samples can be considered “aberrant”. On the basis of the upper 99%CI for normal breast tissue samples (99%CI upper limit ~2% methylation), the incidence of ALKBH3 promoter methylation in primary breast tumors is approximately 27%, i.e. 72 out of 265 primary breast tumors show aberrant ALKBH3 promoter methylation.

RNA samples available from normal breast tissue and tumor samples were used to assess ALKBH3 mRNA expression. Firstly, this analysis demonstrates statistically significant differences in ALKBH3 mRNA expression between normal breast tissue and tumor samples wherein breast tumors generally show reduced ALKBH3 expression (Fig. 4b). This provides an independent confirmation for the previous observations based on the use of available data from the TCGA project shown in Fig. 1b. Secondly, by using DNA samples available from a subset of the same tumor samples as analysed with respect to ALKBH3 expression, we were able to assess the association between mRNA expression levels and promoter methylation status. This analysis, shown in Fig. 4c, further validates the impact of ALKBH3 promoter methylation on mRNA expression levels.

### ALKBH3 promoter methylation with respect to clinical relevance

**Fig. 5:**
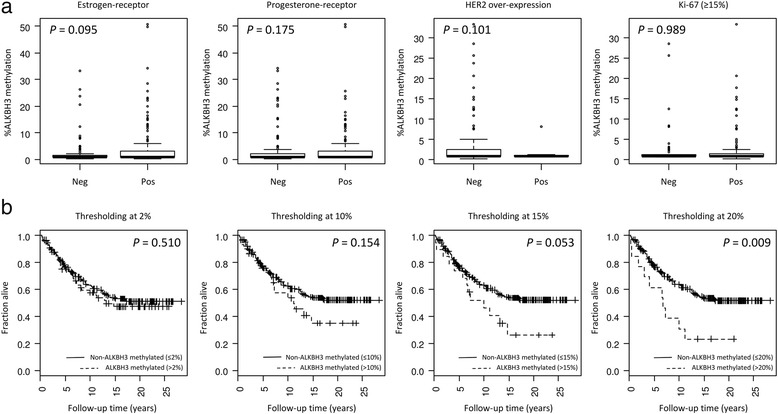
ALKBH3 promoter methylation with respect to clinical parameters and breast cancer-specific survival. **a** ALKBH3 promoter methylation analysed with respect to clinical and subtype-specific markers: estrogen-receptor, progesterone-receptor, HER-2 over-expression and Ki-67. The *P*-values shown were derived from Wilcoxon’s hypothesis testing. **b** ALKBH3 promoter methylation analysed with respect to patient survival (breast cancer-specific survival). The analyses shown were carried out using increasingly higher threshold levels for ALKBH3 promoter methylation as indicated. The *P*-values were derived from log-rank hypothesis testing for differences in survival outcomes

Table 1 displays clinical and pathological characteristics of the patient cohort (*n* = 265). ALKBH3 promoter methylation was not found to be significantly associated with the expression of subtype-specific markers, i.e. estrogen-receptor (ER), progesterone-receptor (PR), erb-b2 receptor tyrosine kinase 2 (known as HER2) and MKI67 (known as Ki-67) as shown in Fig. 5a. Additionally, no associations were found for ALKBH3 promoter methylation in relation to discrete subtype classification based on these four subtype-specific markers, histological grade, tumor size or nodal status (Additional file 3).

**Table 1:** Clinical and pathological characteristics of the patient cohort

	*Positive*	*Negative*		
Estrogen-Receptor	175 (72%)	68 (28%)		
Progestrone-Receptor	136 (56%)	108 (44%)		
HER2-positive (over-expressed)	20 (14%)	121 (86%)		
Ki67-positive (>14% positivity)	84 (60%)	55 (40%)		
	*1986–1991*	*1991–1996*	*1996–2001*	*2001–2004*
Year of Diagnosis	81 (30%)	121 (46%)	60 (23%)	3 (1%)
	*26–42*	*42–58*	*58–74*	*74–91*
Age at Diagnosis	36 (14%)	104 (39%)	80 (30%)	45 (17%)
	*5–30*	*30–55*	*55–80*	*80–100*
Tumour size (mm)	94 (77%)	24 (20%)	1 (1%)	3 (2%)
Nodal status	112 (64%)	63 (36%)		
	*+*	*++*	*+++*	
Histological Grade	19 (13%)	62 (41%)	69 (46%)

Nonetheless, we found significantly reduced survival in patients with tumors showing ALKBH3 promoter methylation (Fig. 5b). This was seen in breast tumors with high cytosine methylation levels over the ALKBH3 promoter region, i.e. those showing at least 20% cytosine methylation (Fig. 5b). This level of methylation is indeed substantially higher than the 2% threshold level defining aberrant CpG methylation as was established by looking at the distribution seen in normal breast tissue samples (see section 3.4).

The incidence of clinically relevant ALKBH3 methylation (thresholding at the 20% cytosine methylation level) is approximately 5% in our cohort (13 of 265; 4.9%) and, on the basis of Cox’s proportional hazards regression analysis, we found that these patients were at approximately 2.3-fold increased risk of death resulting from breast cancer in a multivariate model including adjustment for age and year at diagnosis (HR = 2.3; *P* = 0.012).

## Discussion

### ALKBH3 repression by epigenetic mechanisms in breast cancer

In this study, we show that the ALKBH3 gene promoter region undergoes aberrant epigenetic repression in a significant proportion of primary breast tumours. The ALKBH3 gene is therefore a novel addition to the catalogue of DNA repair genes found inactivated in breast cancer. Previous studies have shown that treatment with the DNA alkylating agent MMS induces the expression of ALKBH3, highlighting the important role of ALKBH3 in repair of alkylation DNA damage [21]. Indeed, our results indicate that inactivation of ALKBH3 is associated with an increased burden of unrepaired alkylation DNA damage involving 3-methylcytosine (3meC) suggesting that our findings has biological consequences. If left unrepaired these modifications can lead to alkylation-induced cell death or can be converted into mutations through the use of error-prone translesion DNA polymerases such as Pol η (eta), Pol ι (iota), and Pol κ (kappa) [31, 32]. Collectively, our observations support the hypothesis that defective repair of alkylation damage occurs in the development of a substantial fraction of breast cancers.

Our results furthermore emphasize the importance of quantification-based methods for DNA methylation analyses in clinical applications. This becomes clear by looking at varied levels of CpG methylation over the ALKBH3 promoter with respect to survival outcomes. Here, increasingly higher levels of promoter methylation were associated with shortened patient survival. Declaring tumours as either ALKBH3 promoter methylated or unmethylated is therefore not a straightforward task. In this study, two different methods are applied for this purpose, i.e. 1) making use of normal tissue samples as reference to then define “abnormally” high levels of ALKBH3 promoter methylation in tumours and 2) by identifying clinically relevant levels of ALKBH3 promoter methylation. Making use of normal breast samples as a reference, does not necessarily provide a means to identify tumours showing ALKBH3 inactivation events. This is because only slightly elevated, but still “abnormal”, promoter methylation might simply reflect passenger events of no relevance to the course of disease progression. The second method refers to making use of clinical parameters involving tumour phenotype or disease survival to then declare a “clinically relevant” threshold for defining ALKBH3 promoter methylated tumours. Given the observed impact on disease progression, this second method is more likely to hold relevant at least as a proxy for identifying tumours affected by ALKBH3 promoter methylation. Using this definition, we report the incidence of ALKBH3 promoter methylation at approximately 5% in our cohort (13 out of 265 primary breast cancers).

### DNA repair deficiency in breast cancer

Defective DNA repair capacity is frequently observed in various human cancers [14]. In breast cancer, this mostly involves defective repair of DNA double-stranded breaks by homologous recombination (HR), e.g. as is known to occur in tumor cells arising in carriers of either BRCA1 or BRCA2 germline mutations [33]. Other potential sources of DNA repair deficiency in breast cancer, also involving DSB repair processes, include germline mutations in DNA repair genes PALB2, FANCM, ATM, CHEK2 (and possibly also RAD51C) characterized as loss-of-function variants [27, 34, 35]. Additionally, somatic mutations occur in both BRCA1 and BRCA2 as well as the ATM gene accounting for a small fraction of patients [27].

In addition to genetic mutations, our previous studies along with others have shown that the BRCA1 gene undergoes CpG promoter methylation in at least 5–10% of all sporadically arising breast tumors [6]. More recently, RAD51C (a RAD51 paralog involved in double-strand break repair) and the RAD51 recombinase have been identified as targets of epigenetic silencing in breast cancer [8, 36, 37]. Other contributors include deregulated expression of miRNAs including miR-182 and miR-146a/b targeting BRCA1 mRNA transcripts for degradation [38, 39].

In summary, the development of breast cancer is clearly linked to inactivation of genes involved in the repair of DNA double-stranded breaks by HR. The involvement of other DNA repair pathways in breast cancer development is, however, currently unclear. Of these, DNA repair of alkylation damage by direct reversal has been suggested following the identification of CpG promoter methylation of the MGMT gene in breast cancer [17]. Our results support this notion by identifying the ALKBH3 gene as a novel addition to the catalogue of DNA repair genes found inactivated in breast cancer.

### The functional consequences of ALKBH3 epigenetic silencing

According to our results, ALKBH3 represents a candidate tumor suppressor gene. A high burden of mutations, caused by ineffective DNA repair, is generally accepted as an important factor in cancer development. Incomplete removal of alkyl groups on DNA has been shown to cause DNA damage, cell cycle arrest and apoptosis. Indeed, cancer genome sequencing has already established an important role for alkylation damage leading to genetic mutations [20]. Consequently, it seems likely that ALKBH3 mediates its tumor suppressive function via its role in DNA alkylation repair. This is supported by the increased level of alkylation damage in ALKBH3 inactivated cells, reported here and in previous studies [30]. It can, however, not be ruled out that other described functions of ALKBH3 also contribute to its anti-tumor activities – including alkylation repair of RNA and the recently described link to regulation of 1-methyladenine mark in mRNA [29, 40–42].

While our data suggest that ALKBH3 has tumor suppressor properties in breast cancer, other research has shown ALKBH3 overexpression in various cancer types. ALKBH3 overexpression is found in prostate cancer [43] and other cancer types [44–48]. In this context, ALKBH3 overexpression likely relates to adaptation of cancer cells to tolerate endogenous alkylation damage to DNA or RNA. This interpretation is in fact relevant in the context of a recent pre-clinical study showing resistance to alkylating drug temozolomide following experimental overexpression of MGMT [49]. Similar results have also been described with respect to ALKBH2 [50].

### Defective repair of alkylation damage and precision medicine

Precision medicine is aimed at optimizing treatment benefits by looking at each patient in terms of genomic or epigenomic variants and, on the basis of this information, to then select the most appropriate combination of cytotoxic or targeted drugs. This is highly relevant with respect to CpG promoter methylation of the MGMT gene now widely recognized as a predictor for patient response to alkylating agents such as temozolomide [19, 51]. Indeed, temozolomide induces methylation damage in DNA involving O6-methylguanine along with N7-methylguanine. In this way, tumor cells lacking the MGMT repair gene as a result of CpG promoter methylation events are highly sensitive to temozolomide [19]. Similarly, in breast cancer, loss of MGMT was recently linked to temozolomide sensitivity in a pre-clinical study [49]. Whether the same principle can be applied with respect to the ALKBH3 gene, i.e. by inducing 3meC and 1meA in patients having developed breast tumors with ALKBH3 promoter methylation, remains to be determined.

## Conclusions

We propose here that the ALKBH3 gene is a novel addition to the catalogue of DNA repair genes found inactivated in breast cancer. The value of ALKBH3 promoter methylation as a prognostic marker was revealed through quantification of CpG methylation levels by pyrosequencing. In this way, our results emphasize the use of quantification-based methods for the assessment of CpG methylation marks in clinical applications. In our cohort, clinically relevant ALKBH3 promoter methylation occurs in at least 5% of all breast cancers and, although independent cohorts will be needed for confirmation, this event appears to be associated with highly aggressive disease behaviour. These observations underscore defective repair of alkylation damage occurring in the development of breast cancer.

## Additional files

Additional file 1: The relation between CpG methylation and expression for the ALKBH3 gene shown here based on available data from the Cancer Genome Atlas project. This catalogue includes only CpG’s found differentially methylated between breast cancers and normal breast tissue samples. The table lists out statistically significant CpG’s with rho < −0.30 and at least two-fold change in expression between unmethylated and methylated tumours. (XLSX 10 kb)

Additional file 2: RNAi for ALKBH3 analysed with respect to 3-methyl-cytosine immunostaining. A) U2OS cells were transfected with a control (scrambled siRNA) and ALKBH3 siRNA for 72 h. After fixation cells were denatured in 1.5 M HCL for 30 min (to gain access to single stranded DNA) and immunostained for 3-me-C and 5-me-C. As expected, decreased ALKBH3 expression resulted in increased accumulation of 3-me-C, indicating less efficient repair, without any detectable changes in 5-me-C levels. B) Quantification of at least 100 cells reveals approximately 1, 6-fold differences in 3-me-C damages based on nuclear intensity measured using CellProfiler. The fold differences are computed as the average signal derived form ALKBH3 siRNA treated cells divided by the average signal derived from scrambled siRNA treated cells (by default set to one in the figure). 3-me-C = 3-methyl-cytosine, 5-me-C = 5-methyl-cytosine. (TIFF 3866 kb)

Additional file 3: ALKBH3 methylation analysed with respect to prognostic parameters (breast cancer subtype, histological grade, tumour size and nodal status). (XLSX 12 kb)

## Abbreviations

1meA: 1-methyladenine
3meC: 3-methylcytosine
5meC: 5-methylcytosine
ALKBH3: alkB homolog 3, alpha-ketoglutarate-dependent dioxygenase
MMS: Methyl methanesulfonate
qPCR: Quantitative Polymerase Chain Reaction
TCGA: The Cancer Genome Atlas
vHMEC: Variant human mammary epithelial cells

## References

[1] Cutter AR, Hayes JJ (2015). A brief Review of nucleosome structure. FEBS Lett.

[2] Hesson LB, Sloane MA, Wong JW, Nunez AC, Srivastava S, Ng B (2014). Altered promoter nucleosome positioning is an early event in gene silencing. Epigenetics.

[3] Jones PA (2012). Functions of DNA methylation: islands, start sites, gene bodies and beyond. Nat Rev Genet.

[4] Baylin SB, Jones PA (2011). A decade of exploring the cancer epigenome - biological and translational implications. Nat Rev Cancer.

[5] Simó-Riudalbas L, Esteller M (2014). Cancer genomics identifies disrupted epigenetic genes. Hum Genet.

[6] Stefansson OA, Jonasson JG, Olafsdottir K, Hilmarsdottir H, Olafsdottir G, Esteller M (2011). CpG island hypermethylation of BRCA1 and loss of pRb as co-occurring events in basal/triple-negative breast cancer. Epigenetics.

[7] Stefansson OA, Moran S, Gomez A, Sayols S, Arribas-Jorba C, Sandoval J (2015). A DNA methylation-based definition of biologically distinct breast cancer subtypes. Mol Oncol.

[8] Cunningham JM, Cicek MS, Larson NB, Davila J, Wang C, Larson MC (2014). Clinical characteristics of ovarian cancer classified by BRCA1, BRCA2, and RAD51C status. Sci Rep.

[9] Muggerud AA, Rønneberg JA, Wärnberg F, Botling J, Busato F, Jovanovic J (2010). Frequent aberrant DNA methylation of ABCB1, FOXC1, PPP2R2B and PTEN in ductal carcinoma in situ and early invasive breast cancer. Breast Cancer Res.

[10] Subramaniam MM, Chan JY, Soong R, Ito K, Ito Y, Yeoh KG (2009). RUNX3 inactivation by frequent promoter hypermethylation and protein mislocalization constitute an early event in breast cancer progression. Breast Cancer Res Treat.

[11] Lalloo F, Evans DG (2012). Familial breast cancer. Clin Genet.

[12] Collins N, Wooster R, Stratton MR (1997). Absence of methylation of CpG dinucleotides within the promoter of the breast cancer susceptibility gene BRCA2 in normal tissues and in breast and ovarian cancers. Br J Cancer.

[13] Brandes JC, Carraway H, Herman JG (2007). Optimal primer design using the novel primer design program: MSPprimer provides accurate methylation analysis of the ATM promoter. Oncogene.

[14] Lord CJ, Ashworth A (2016). BRCAness revisited. Nat Rev Cancer.

[15] Saal LH, Gruvberger-Saal SK, Persson C, Lövgren K, Jumppanen M, Staaf J (2008). Recurrent gross mutations of the PTEN tumor suppressor gene in breast cancers with deficient DSB repair. Nat Genet.

[16] Spitzwieser M, Holzweber E, Pfeiler G, Hacker S, Cichna-Markl M (2015). Applicability of HIN-1, MGMT and RASSF1A promoter methylation as biomarkers for detecting field cancerization in breast cancer. Breast Cancer Res.

[17] Fumagalli C, Pruneri G, Possanzini P, Manzotti M, Barile M, Feroce I (2012). Methylation of O6-methylguanine-DNA methyltransferase (MGMT) promoter gene in triple-negative breast cancer patients. Breast Cancer Res Treat.

[18] Drabløs F, Feyzi E, Aas PA, Vaagbø CB, Kavli B, Bratlie MS (2004). Alkylation damage in DNA and RNA--repair mechanisms and medical significance. DNA Repair (Amst).

[19] Esteller M, Garcia-Foncillas J, Andion E, Goodman SN, OF H, Vanaclocha V (2000). Inactivation of the DNA-repair gene MGMT and the clinical response of gliomas to alkylating agents. N Engl J Med.

[20] Alexandrov LB, Nik-Zainal S, Wedge DC, Aparicio SA, Behjati S, Biankin AV (2013). Signatures of mutational processes in human cancer. Nature.

[21] Sedgwick B, Bates PA, Paik J, Jacobs SC, Lindahl T (2007). Repair of alkylated DNA: recent advances. DNA Repair (Amst).

[22] Sigurdardottir LG, Jonasson JG, Stefansdottir S, Jonsdottir A, Olafsdottir GH, Olafsdottir EJ, Tryggvadottir L (2012). Data quality at the Icelandic cancer Registry: comparability, validity, timeliness and completeness. Acta Oncol.

[23] Lizio M, Harshbarger J, Shimoji H, Severin J, Kasukawa T, Sahin S (2015). Gateways to the FANTOM5 promoter level mammalian expression atlas. Genome Biol.

[24] Stefansson OA, Jonasson JG, Johannsson OT, Olafsdottir K, Steinarsdottir M, Valgeirsdottir S, Eyfjord JE (2009). Genomic profiling of breast tumors in relation to BRCA abnormalities and phenotypes. Breast Cancer Res.

[25] Dedeurwaerder S, Desmedt C, Calonne E, Singhal SK, Haibe-Kains B, Defrance M (2011). DNA methylation profiling reveals a predominant immune component in breast cancers. EMBO Mol Med.

[26] Teschendorff AE, Gao Y, Jones A, Ruebner M, Beckmann MW, Wachter DL (2016). DNA methylation outliers in normal breast tissue identify field defects that are enriched in cancer. Nat Commun.

[27] Network CGA (2012). Comprehensive molecular portraits of human breast tumors. Nature.

[28] Roadmap Epigenomics Consortium (2015). Integrative analysis of 111 reference human epigenomes. Nature.

[29] Ougland R, Rognes T, Klungland A, Larsen E (2015). Non-homologous functions of the AlkB homologs. J Mol Cell Biol.

[30] Dango S, Mosammaparast N, Sowa ME, Xiong LJ, Wu F, Park K (2011). DNA unwinding by ASCC3 helicase is coupled to ALKBH3-dependent DNA alkylation repair and cancer cell proliferation. Mol Cell.

[31] Goodman MF, Woodgate R (2013). Translesion DNA polymerases. Cold Spring Harb Perspect Biol.

[32] Waters LS, Minesinger BK, Wiltrout ME, D'Souza S, Woodruff RV, Walker GC (2009). Eukaryotic translesion polymerases and their roles and regulation in DNA damage tolerance. Microbiol Mol Biol Rev.

[33] Nik-Zainal S, Davies H, Staaf J, Ramakrishna M, Glodzik D, Zou X, Martincorena I, Alexandrov LB, Martin S, Wedge DC, Van Loo P, Ju YS, Smid M, Brinkman AB, Morganella S, Aure MR, Lingjærde OC, Langerød A, Ringnér M, Ahn SM, Boyault S, Brock JE, Broeks A, Butler A, Desmedt C, Dirix L, Dronov S, Fatima A, Foekens JA, Gerstung M, Hooijer GK, Jang SJ, Jones DR, Kim HY, King TA, Krishnamurthy S, Lee HJ, Lee JY, Li Y, McLaren S, Menzies A, Mustonen V, O’Meara S, Pauporte I, Pivot X, Purdie CA, Raine K, Ramakrishnan K, Rodríguez-González FG, Romieu G, Sieuwerts AM, Simpson PT, Shepherd R, Stebbings L, Stefansson OA, Teague J, Tommasi S, Treilleux I, Van den Eynden GG, Vermeulen P, Vincent-Salomon A, Yates L, Caldas C, Veer LV, Tutt A, Knappskog S, Tan BK, Jonkers J, Borg Å, Ueno NT, Sotiriou C, Viari A, Futreal PA, Campbell PJ, Span PN, Van Laere S, Lakhani SR, Eyfjord JE, Thompson AM, Birney E, Stunnenberg HG, van de Vijver MJ, Martens JW, Børresen-Dale AL, Richardson AL, Kong G, Thomas G, Stratton MR. Landscape of somatic mutations in 560 breast cancer whole-genome sequences. Nature. 2016; doi:10.1038/nature17676.

[34] Ghoussaini M, Pharoah PD, Easton DF (2013). Inherited genetic susceptibility to breast cancer: the beginning of the end or the end of the beginning?. Am J Pathol.

[35] Peterlongo P, Catucci I, Colombo M, Caleca L, Mucaki E, Bogliolo M (2015). FANCM c.5791C&gt;T nonsense mutation (rs144567652) induces exon skipping, affects DNA repair activity and is a familial breast cancer risk factor. Hum Mol Genet.

[36] Chang CJ, Yang JY, Xia W, Chen CT, Xie X, Chao CH (2011). EZH2 promotes expansion of breast tumor initiating cells through activation of RAF1-beta-catenin signaling. Cancer Cell.

[37] Stefansson OA, Esteller M (2011). EZH2-mediated epigenetic repression of DNA repair in promoting breast tumor initiating cells. Breast Cancer Res.

[38] Moskwa P, Buffa FM, Pan Y, Panchakshari R, Gottipati P, Muschel RJ (2011). miR-182-mediated downregulation of BRCA1 impacts DNA repair and sensitivity to PARP inhibitors. Mol Cell.

[39] Garcia AI, Buisson M, Bertrand P, Rimokh R, Rouleau E, Lopez BS (2011). Down-regulation of BRCA1 expression by miR-146a and miR-146b-5p in triple negative sporadic breast cancers. EMBO Mol Med.

[40] Aas PA, Otterlei M, Falnes PO, Vågbø CB, Skorpen F, Akbari M (2003). Human and bacterial oxidative demethylases repair alkylation damage in both RNA and DNA. Nature.

[41] Li X, Xiong X, Wang K, Wang L, Shu X, Ma S, Yi C. Transcriptome-wide mapping reveals reversible and dynamic N1-methyladenosine methylome. Nat Chem Biol. 2016; 10.1038/nchembio.2040 26863410

[42] Dominissini D, Nachtergaele S, Moshitch-Moshkovitz S, Peer E, Kol N, Ben-Haim MS (2016). The dynamic N(1)-methyladenosine methylome in eukaryotic messenger RNA. Nature.

[43] Konishi N, Nakamura M, Ishida E, Shimada K, Mitsui E, Yoshikawa R (2005). High expression of a new marker PCA-1 in human prostate carcinoma. Clin Cancer Res.

[44] Tasaki M, Shimada K, Kimura H, Tsujikawa K, Konishi N (2011). ALKBH3, a human AlkB homologue, contributes to cell survival in human non-small-cell lung cancer. Br J Cancer.

[45] Yamato I, Sho M, Shimada K, Hotta K, Ueda Y, Yasuda S (2012). PCA-1/ALKBH3 contributes to pancreatic cancer by supporting apoptotic resistance and angiogenesis. Cancer Res.

[46] Cetica V, Genitori L, Giunti L, Sanzo M, Bernini G, Massimino M, Sardi I (2009). Pediatric brain tumors: mutations of two dioxygenases (hABH2 and hABH3) that directly repair alkylation damage. J Neuro-Oncol.

[47] Choi SY, Jang JH, Kim KR (2011). Analysis of differentially expressed genes in human rectal carcinoma using suppression subtractive hybridization. Clin Exp Med.

[48] Shimada K, Fujii T, Tsujikawa K, Anai S, Fujimoto K, Konishi N (2012). ALKBH3 contributes to survival and angiogenesis of human urothelial carcinoma cells through NADPH oxidase and tweak/Fn14/VEGF signals. Clin Cancer Res.

[49] Palmieri D, Duchnowska R, Woditschka S, Hua E, Qian Y, Biernat W (2014). Profound prevention of experimental brain metastases of breast cancer by temozolomide in an MGMT-dependent manner. Clin Cancer Res.

[50] Johannessen TC, Prestegarden L, Grudic A, Hegi ME, Tysnes BB, Bjerkvig R (2013). The DNA repair protein ALKBH2 mediates temozolomide resistance in human glioblastoma cells. Neuro-Oncology.

[51] Weller M, Tabatabai G, Kästner B, Felsberg J, Steinbach JP, Wick A (2015). MGMT promoter Methylation is a strong prognostic biomarker for benefit from dose-intensified Temozolomide Rechallenge in progressive Glioblastoma: the DIRECTOR trial. Clin Cancer Res.

